# Selecting informative subsets of sparse supermatrices increases the chance to find correct trees

**DOI:** 10.1186/1471-2105-14-348

**Published:** 2013-12-03

**Authors:** Bernhard Misof, Benjamin Meyer, Björn Marcus von Reumont, Patrick Kück, Katharina Misof, Karen Meusemann

**Affiliations:** 1, Zoologisches Forschungsmuseum Alexander Koenig, zmb, Adenauerallee 160, 53113 Bonn, Germany; 2 Institut für Systematische Neurowissenschaften, Universitätsklinikum Hamburg Eppendorf, Martinistr. 52, 20246 Hamburg, Germany; 3 Natural History Museum, London Department of Life Sciences, Cromwell Road, London, SW7 5BD, UK; 4 CSIRO Ecosystem Sciences, Australian National Insect Collection, Clunies Ross Street, Acton, ACT, Australia

## Abstract

**Background:**

Character matrices with extensive missing data are frequently used in phylogenomics with potentially detrimental effects on the accuracy and robustness of tree inference. Therefore, many investigators select taxa and genes with high data coverage. Drawbacks of these selections are their exclusive reliance on data coverage without consideration of actual signal in the data which might, thus, not deliver optimal data matrices in terms of potential phylogenetic signal. In order to circumvent this problem, we have developed a heuristics implemented in a software called *mare *which (1) assesses information content of genes in supermatrices using a measure of potential signal combined with data coverage and (2) reduces supermatrices with a simple hill climbing procedure to submatrices with high total information content. We conducted simulation studies using matrices of 50 taxa × 50 genes with heterogeneous phylogenetic signal among genes and data coverage between 10–30%.

**Results:**

With matrices of 50 taxa × 50 genes with heterogeneous phylogenetic signal among genes and data coverage between 10–30% Maximum Likelihood (ML) tree reconstructions failed to recover correct trees. A selection of a data subset with the herein proposed approach increased the chance to recover correct partial trees more than 10-fold. The selection of data subsets with the herein proposed simple hill climbing procedure performed well either considering the information content or just a simple presence/absence information of genes. We also applied our approach on an empirical data set, addressing questions of vertebrate systematics. With this empirical dataset selecting a data subset with high information content and supporting a tree with high average boostrap support was most successful if information content of genes was considered.

**Conclusions:**

Our analyses of simulated and empirical data demonstrate that sparse supermatrices can be reduced on a formal basis outperforming the usually used simple selections of taxa and genes with high data coverage.

## Background

In most phylogenomic studies supermatrices of concatenated presumably orthologous genes are used for tree inference [1-18]. Due to the failure of consistently identifying orthologous genes among taxa [2] and/or due to general sparse sequence data availability these supermatrices frequently display a low data coverage, down to 8% [2]. Simulation studies showed that in these instances chances of recovering a correct and robust tree can drastically decrease [1,19]. Additionally, Wiens [20,21], Philippe [22], Sanderson [1,19,23], Driskell [2], Hartmann [24] and colleagues showed that low gene data coverage of single taxa can already impede the success of tree reconstructions. In contrast, other simulation studies demonstrated that single taxa with low gene data coverage can help breaking up long branches and thus improve tree reconstructions [20,21,25-28]. These mentioned analyses of empirical and simulated data demonstrate that confounding effects of low gene data coverage on tree inference can hardly be generalized [1,3,11,29-36].

Despite these unresolved issues many investigators select sets of taxa with high gene data coverage assuming that the high gene data coverage will improve the robustness of tree inferences [3,4,9,11,16,17]. However, these threshold criteria are arbitrary and do not take into account potential phylogenetic signal of the data. Those approaches might not lead to the desired increase of tree robustness. For example, tree robustness will not increase, if high gene data coverage is achieved by selecting highly conservative orthologous genes with low phylogenetic signal. Alternatively, a robust tree might result if taxa with low gene data coverage but highly informative genes have been selected, Driskell et al. [2] e.g. report an example of plausible tree reconstructions based on a supermatrix with a gene data coverage of just 8–16%. Both cases illustrate that gene data coverage and phylogenetic resolution are not necessarily correlated. Consequently, the practice of selecting data based solely on data coverage is potentially problematic. Therefore, we have developed an approach which focuses on the analyses of **s**elected **o**ptimal data **s**ubsets (SOS) which have high data coverage *and* phylogenetic signal. Crucial for this approach is the assessment of potential signal of genes and the development of a heuristics to select such an SOS.

Different quartet mapping approaches have been used to assess potential signal within genes [37,38]. Among these, geometry mapping is demonstrably the most conservative estimator [37] and the application to genes of supermatrices is straightforward. Consequently, we have chosen the geometry mapping approach [37-40] to assess potential signal of genes in the development of our heuristics.

In order to select an optimal set of taxa and genes, Sanderson and colleagues [23] suggested selecting sets of full data coverage (*maximal bicliques *[41,42]). However, the identification of the *maximal (maximum) biclique* is a NP-complete problem [42,43] and, thus, there is no guarantee to find the *maximal (maximum) biclique*. Additionally, Sanderson et al. [23] found that selections of *maximal bicliques* resulted in very small subsets of size < 15 taxa and < 10 genes. Sanderson’s approach is, thus, not suitable to reconstruct phylogenetic relationships of many taxa. A possible solution might be the selection of *quasi-bicliques *[44,45], which potentially combine a much larger set of taxa and genes accepting a predefined level of missing data. This promising direction however has the drawback that it is not time-efficient.

Alternatively Hartmann et al. [24] and Cheng et al. [46] introduced two approaches directly applicable to sequence data. The first approach of Hartmann et al. [24] is a masking technique (REAP) which masks multiple sequence alignments according to predefined thresholds of gap frequencies of sites. The approach of Cheng et al. [46] is a statistical correction for missing data (SIA). A comparison of these two approaches demonstrated that REAP performed better, a result which is compatible with the results of Sanderson’s *biclique* approach. However, both, alignment masking (REAP) and the *biclique* approach optimize data only with respect to data coverage and without considering potential signal among genes.

Here, we introduce a simple hill climbing algorithm to select optimal data subsets (SOS) which are assembled by considering data coverage and potential signal of genes. We start with the assumption that any taxon and gene can potentially contribute to the total signal of the matrix. However, taxa or genes with incomplete data coverage and low signal can potentially also contribute noise or cause biases to the total signal of the supermatrix. Therefore, we successively mask taxa and genes of low signal and/or data coverage generating a submatrix of higher data coverage and signal. With this approach we deliberately discard taxa and genes because of their low data coverage and/or potential low signal. The proposed hill climbing algorithm delivers an optimal solution of this trade-off. Using simulated and empirical data, we compare the performance of the herein proposed approach with an often applied approach of simply selecting data subsets using predefined thresholds of data coverage only.

## Methods

The approach can be separated into two parts: (1) the determination of information content of genes, taxa and the concatenated supermatrix and (2) the selection of an optimal subset (SOS) of taxa and genes.

### Information content of genes, taxa and matrices

Before we define the *information content of genes*, *taxa* and *matrices* used in our approach, we have to introduce the concepts of *data coverage representation matrices*.

A concatenated supermatrix of *N* taxa and *n* gene nucleotide/amino acid sequence alignments can be represented as a matrix *B* with entries *b*_*ij *_

(1) $$B:b_{\text{ij}}=(1|0),\forall (\text{taxa}:i:1\ldots N,\text{genes}:j:1\ldots n)$$

with *b*_*ij *_= (1) for a present and *b*_*ij *_= (0) for an absent gene nucleotide/amino acid sequence *j* for a taxon *i*. We call this matrix *B* the *data coverage representation matrix*.

We define the information content of a gene *j*, *q*_*j*_, as the relative data coverage of this gene, defined as

(2) $$q_{j}=\frac{\overset{N}{\underset{i=1}{\sum }}b_{\text{ij}}}{N},\forall \text{taxa}:i:1\ldots \mathrm{N.}$$

Likewise, the information content of a taxon *i*, *p*_*i *_is defined as

(3) $$p_{i}=\frac{\overset{n}{\underset{j=1}{\sum }}b_{\text{ij}}}{n},\forall \text{genes}:j:1\ldots \mathrm{n.}$$

**Figure 1:**
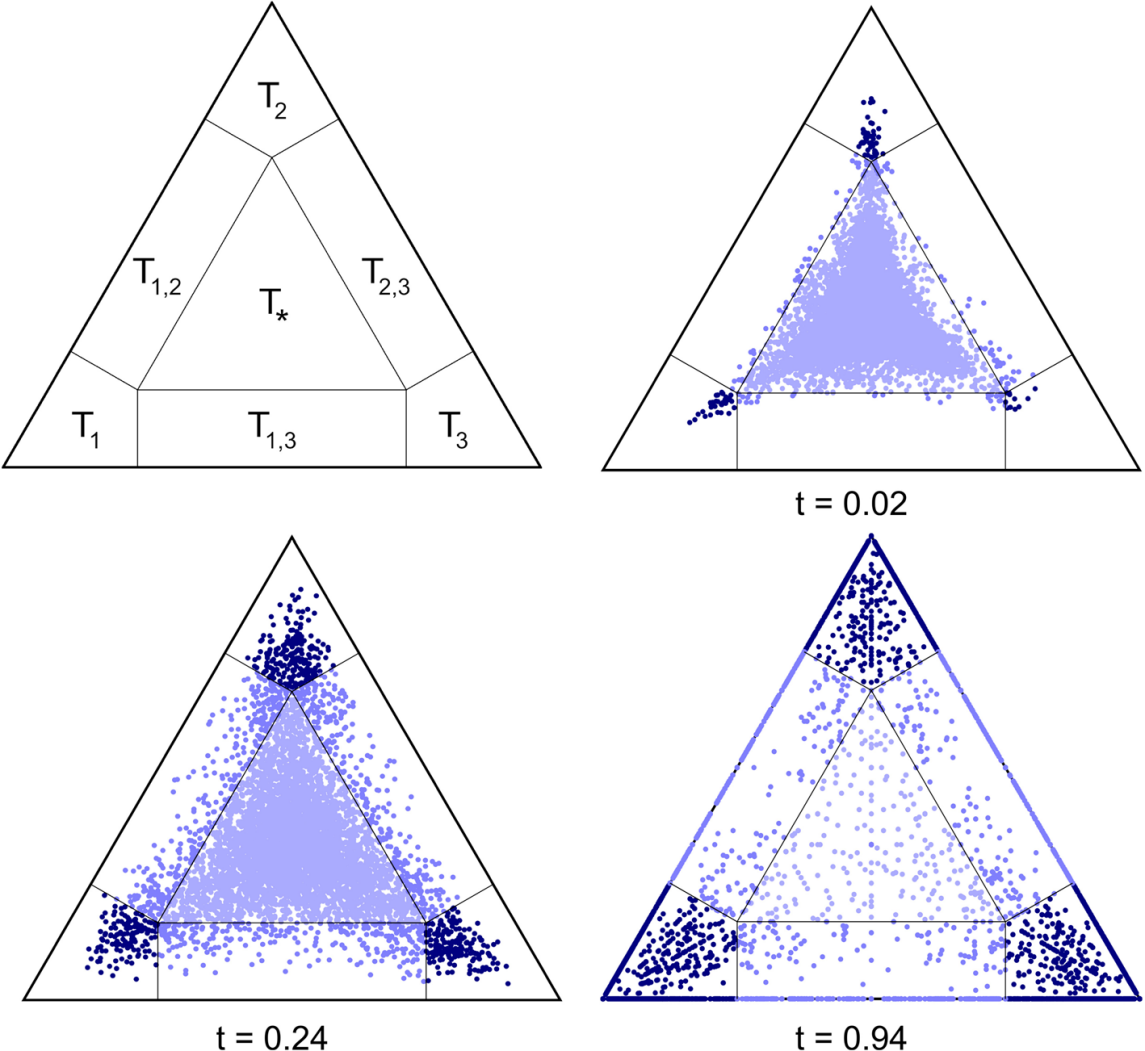
**Simplex graphs of three different partitions of simulated data.** The area of *ϕ*_∗_ corresponds to the inner triangle in light blue, the area of *ϕ*_*r*_ to the surrounding region. Attractors of topologies *T*_1_,*T*_2_,*T*_3_ are found in the corner sections of the outer triangle in dark blue.

We define the information content, *P*, of a matrix *B* as

(4) $$P(B)=\frac{\overset{N}{\underset{i=1}{\sum }}\overset{n}{\underset{j=1}{\sum }}p_{i}}{N\times n}=\frac{\overset{N}{\underset{i=1}{\sum }}\overset{n}{\underset{j=1}{\sum }}q_{j}}{N\times n}$$

with 0 ≤ *P *(*B*),*p*_*i*_,*q*_*j *_≤ 1. To determine the potential signal of genes we use geometry mapping [37] extended to the amino acid level. Nieselt-Struwe et al. [37] showed that for a given quartet of sequences, relative support for each of the three possible topologies *s*_1_,*s*_2_,*s*_3_ can be computed as

(5) $$s_{i}=\delta_{i}/(\delta_{1}+\delta_{2}+\delta_{3})$$

with *δ*_*i *_support for tree *T*_*i*_, 0 ≤ *s*_*i *_≤ 1 and $\underset{i}{\sum }s_{i}=1$. Support values *δ*_*i *_can be computed with any optimality criterion. Relative support values can be interpreted as baricentric coordinates of a bipartite simplex graph *S * with vectors *s *= (*s*_1_,*s*_2_,*s*_3_):

(6) $$S=\left\{\overset{3}{\underset{i=1}{\sum }}s_{i}e_{i}|s_{1}+s_{2}+s_{3}=1,0\leq s_{1},s_{2},s_{3}\leq 1\right\}$$

with *e*_*i *_as unit vectors. Within *S*, areas *T*_1_,*T*_2_,*T*_3_ at vertices can be defined for resolved quartets, *T*_1,2_,*T*_1,3_,*T*_2,3_ for partly resolved quartets, and *T*_∗_ for star-like, unresolved topologies of quartets [37, see Figure 1 ]. For all possible quartets *k*_*j *_of a gene *j*, kj=N4 with *N* the number of taxa, all vectors *s*_*m *_= (*s*_1_,*s*_2_,*s*_3_),(∀ *m *: 1 … *k*) can be calculated, and the frequency of vectors in areas *T*_1_,*T*_2_, and *T*_3_ determine potential signal, *t*_*j *_of a gene *j*[37].

(7) $$t_{j}=\frac{T_{1}+T_{2}+T_{3}}{T_{1}+T_{2}+T_{3}+T_{1,2}+T_{1,3}+T_{2,3}+T_{*}}$$

We relaxed the definition of signal by calculating the frequency of vectors in areas *T*_1_,*T*_2_,*T*_3_,*T*_1,2_,*T*_1,3_,*T*_2,3_.

(8) $$\hat{t_{j}}=\frac{T_{1}+T_{2}+T_{3}+T_{1,3}+T_{2,3}+T_{1,2}}{T_{1}+T_{2}+T_{3}+T_{1,2}+T_{1,3}+T_{2,3}+T_{*}}$$

Our approach will, thus, be a more optimistic estimator of potential signal. Signal $\hat{t_{j}}$ will be $0\leq \hat{t_{j}}\leq 1$ (examples of simulated data, Figure 1).

Geometry mapping is a conservative estimator of $\hat{t_{j}}$, however, within a narrow range of short internal and long terminal branch lengths, geometry mapping opts for the wrong tree, a classical case of long branch attraction [37]. This phenomenon might inflate the estimation of $\hat{t_{j}}$ under certain circumstances.

Nieselt-Struwe and colleagues [37] showed that for any alphabet of characters of finite length, e.g. nucleotides or amino acids, an enumeration of character states among four sequences can be used to calculate support for all three possible topologies. They further showed that a weight matrix *M*, defining dissimilarity measures between characters, can equivalently be used to calculate distances between sequences. Therefore, we used BLOSUM62, the amino acid substitution matrix introduced by Henikoff [47], to calculate distances between sequences in correspondence to equation (8) in Nieselt-Struwe et al. [37].

We use $\hat{t_{j}}$ of each gene *j* to update entries of matrix *B*. For each gene *j*, entries of matrix *B *= (*b*_*ij*_) are scaled with the corresponding $\hat{t_{j}}$ values. We call this matrix a *weighted data coverage representation matrix B*^∗^, in short, a *weighted matrix B*^∗^, in the following:

(9) $$\begin{array}{ll} B^{*}:b_{\text{ij}}^{*}= & \;(0\leq b_{\text{ij}}\hat{t_{j}}\leq 1),\\ & \;\forall (\text{taxa}:i:1\ldots N,\text{genes}:j:1\ldots n) \end{array}$$

Substituting $b_{\text{ij}}^{*}$ for *b*_*ij *_results in weighted forms of equations 1 and 2. The information content of a gene *j*, *q*_*j*_, represents in its weighted form a product of relative data coverage and potential signal of genes.

### Selection of an optimal subset (SOS) of taxa and genes

We consider a subset(=submatrix) of taxa and genes optimal, if it has a high information content, *P *(*B*) and contains as many taxa and genes as possible. If we discard genes or taxa with low *q*_*j *_or *p*_*i *_respectively, we will increase *P* of the matrix, but will loose information on the excluded taxa and genes. A simple optimization can be performed, searching for the highest possible *P* while excluding as few taxa/genes as possible.

First, a data coverage representation matrix *B* is generated from the concatenated supermatrix of multiple gene nucleotide/amino acid sequences corresponding to equation (1). Secondly, for each gene *j*, ≤20,000 quartets are randomly drawn without duplication and $\hat{t_{j}}$ is calculated. For each gene *j*, entries of *B *= (*b*_*ij*_) are scaled with the corresponding $\hat{t_{j}}$ values, generating a weighted matrix *B*^∗^ corresponding to equation (6). Thirdly, we use a simple hill climbing procedure to select an optimal subset (SOS) of taxa and genes. Elimination of taxa or genes starts with dropping either a taxon or gene with the lowest information content *p*_*i *_or *q*_*j*_, generating a new matrix *B*^′^ with *P*^′ ^(*B*^′^). In case of ties between *q*_*j *_and *p*_*i*_, genes will be excluded. Since taxa or genes with lowest information content will be dropped, *P*^′ ^(*B*^′^) > *P *(*B*) (it is trivial to show that this will always be the case). After each elimination step, information content of taxa (*p*_*i*_) and genes (*q*_*j*_) are recalculated. Every gene represented by less than 4 taxa is automatically dropped from the matrix. Gene overlap between taxa is monitored to a minimum of three taxa and two genes. If the matrix *B*^′^ does not fulfill this criterion, the next best *B*^′^ in terms of *P*^′^ is selected.

Continuous elimination of taxa or genes with low *p*_*i*_ or *q*_*j *_will generate a ‘trivial’ SOS containing few taxa and one gene. Therefore, we define an optimality function *f *(*P*)

(10) $$f(P)=1-|(\lambda -P^{\alpha \times (1-P)})|\text{if}\;P<1$$

with *α* as a scaling factor (default set to *α *= 3) and *λ* as the size ratio between reduced *B*^′^ and original matrix *B*

(11) $$\lambda =\frac{N_{B^{\prime }}\times n_{B^{\prime }}}{N_{B}\times n_{B}}.$$

**Figure 2:**
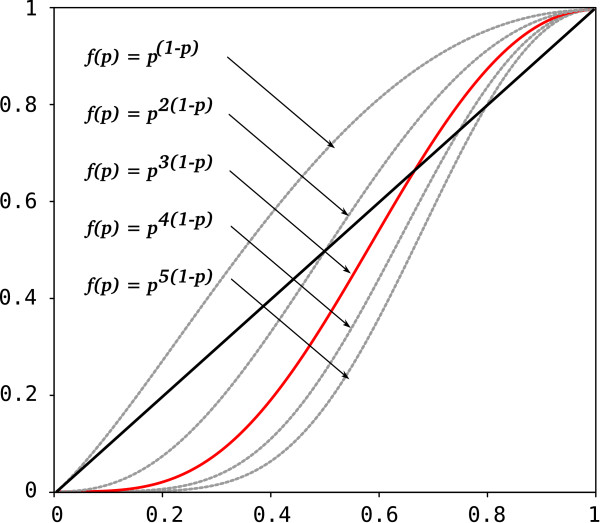
Optimality function and its effects.

**Figure 3:**
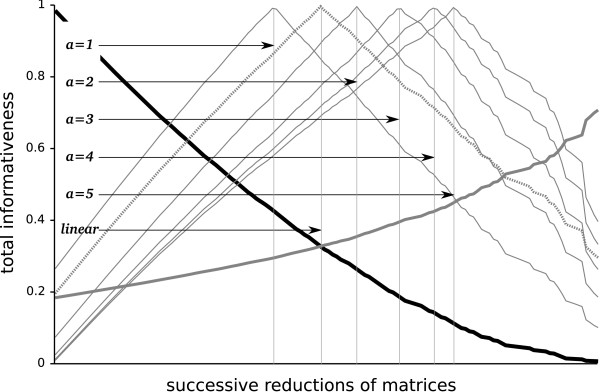
**Influence of different *α *values on the identification of reduction optima in simulated data matrices.** The dark bold descending line corresponds to *λ*, size reduction; the grey bold ascending line to the increase in total informativeness.

During the process of elimination of taxa and/or genes, *P*^′^ will continually increase, and *λ* will continually decrease. *f*(*P*^′^) will reach a maximum of 1. With a scaling factor *α *= 2, the maximum will be at the intersection of *P*^′^ and *λ*, with *α *= 3 it will be reached later, favoring an SOS with a higher *P* (Figures 2 and 3). If *f *(*P*^′^) = 1 the process of elimination stops.

The outlined procedure is a simple hill climbing heuristics without guarantee of finding a globally optimal solution due to the interaction of *p*_*i *_and *q*_*j*_. The approach can be applied either to *B* or *B*^∗^. It should be pointed out that removal of taxa will have an influence on the calculation of $\hat{t_{j}}$ which is not recalculated during the process of matrix reduction. This simplification greatly speeds up the heuristics. An iterative recalculation of $\hat{t_{j}}$ can potentially improve the selection of an informative dataset and will be further studied.

Calculation time for this heuristics grows with the number of taxa (*N*) and genes (*n*). Therefore, it is time efficient, *O *(*N *+ *n*)^2^. The algorithm reduces matrices in a deterministic way which makes matrix reduction reproducible. However, different equally optimal solutions will not be found under identical parameter settings.

By varying the scaling parameter *α*, however, an SOS of high *P* (*α *≥ 3), versus an SOS of more taxa and genes with lower *P* (*α *≤ 3) can be found.

### Simulated data

Our simulations were not set up with the intention of fully exploring the performance of matrix reductions depending on super matrix characteristics, but were set up in order to illustrate the potential of the method in four different cases, resembling observed situations of empirical data.

#### ***Simulated data with random distribution of missing data***

**Figure 4:**
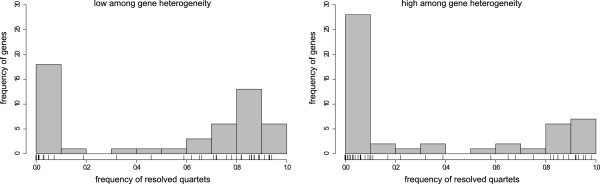
**Histograms of heterogeneity of signal among genes of simulated data.** On the left, set 1, the histogram of simulated data shows relative low heterogeneity of signal among genes, on the right, set 2, the histogram shows relative high heterogeneity of signal among genes, with a higher percentage of genes of low potential information content.

**Figure 5:**
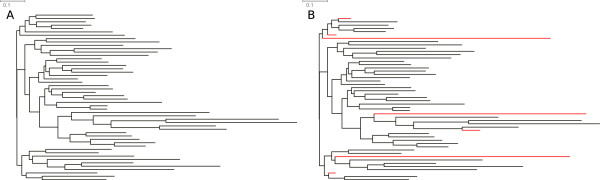
**Topologies with branch lengths used for data simulation.** Different branch lengths between tree **A** and **B** are labeled in light grey in tree **B**.

**Figure 6:**
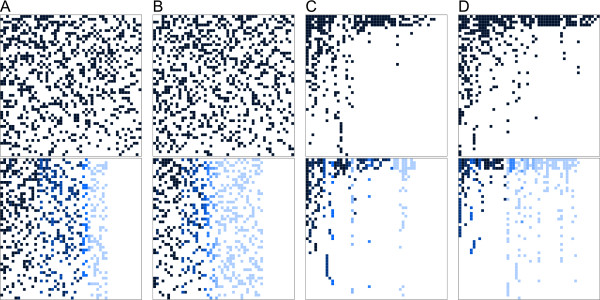
**Examples of presence/absence *B * and edge-weighted *B***^**∗ **^**data used in simulations.** Matrices of data availability in the upper panels, transformed edge-weighted matrices in the lower panels. All matrices are sorted. **(A,B) ** 20% data availability, relative **(A)** low and **(B)** high heterogeneity of potential signal, missing data Gaussian distributed. **(C,D)** 10% data availability, relative **(C)** low and **(D)** high heterogeneity of potential signal, missing data following a power-law non-random distribution.

For two different sets of genes, differing in relative evolutionary rates among genes (Figure 4), we simulated 100 (50 taxa × 50 genes) supermatrices each, composed of genes with 400 amino acids (aa), concatenated for each taxon to 20,000 aa length using Seq-Gen [48] and the BLOSUM62 matrix. For these simulations, we used a topology derived from empirical data with realistic distribution of branch lengths (Figure 5A). Evolutionary rates of genes varied from 0.001 to 15.00 relative rate differences, to mimic different signal strength (Figures 4 and 6). Within each gene, site rates were homogeneous. In order to generate supermartices with missing data, we removed amino acid sequences of taxa using a Binomial distribution with a probability of retaining data entries for each taxon and gene of 0.7 (average data coverage of 0.29, Table 1). This set up generated supermatrices with randomly distributed missing data, closely resembling the observed data coverage of published concatenated supermatrices of Dunn and colleagues [4].

**Table 1:** Summary of simulation results

**Simulation**	**Saturation**	**tic**^**∗**^	**taxa**	**Genes**	***d***_***QS***_**–value**	***d***_***QS***_	***f***
						**(min/max)**	**( *correct ‡ *)**
Gaussian Set1							
Unreduced	0.29	0.15	50	50	0.003	(0.99/1.0)	0.01
*mare *with *B*^∗^	0.69	0.62	9	6	0.0	(0.73/1.0)	0.67
*mare *with *B*	0.74	0.74	7	9	0.0	(0.6/1.0)	0.47
Gaussian Set2							
Unreduced	0.29	0.1	50	50	0.003	(0.98/0.99)	0
*mare *with *B*^∗^	0.67	0.61	10	5	0	(0.6/1.0)	0.51
*mare *with *B*	0.73	0.73	7	9	0	(0.2/1)	0.42
Power-law non-random Set1							
Unreduced	0.13	0.06	50	50	0.17	(0.48/0.99)	0
*mare *with *B*^∗^	0.46	0.38	25	12	0.02	(0.81/1.0)	0.15
*mare *with *B*	0.51	0.51	15	24	0.02	(0.48/1.0)	0.16
Power-law non-random Set2							
Unreduced	0.13	0.05	50	50	0.15	(0.43/0.99)	0
*mare *with *B*^∗^	0.45	0.38	24.5	10	0.06	(0.64/1.0)	0.09
*mare *with *B*	0.53	0.53	23	16	0.01	(0.47/1.0)	0.12
Gene threshold Set1							
With *B*^∗^	0.72	0.50	34	2	0.05	(0.00/0.42)	0.06
Gene threshold Set2							
With *B*	0.64	0.28	44	3	0.03	(0.00/0.59)	0.03
Gene/taxa threshold Set1							
With *B*^∗^	0.59	0.37	21	4	0.05	(0.00/0.46)	0.12
Gene/taxa threshold Set2							
With *B*	0.66	0.30	21.5	4	0.01	(0.00/0.45)	0.25

All values are medians of 100 simulations.

∗ total information content (tic) of un-weighted matrices is allways higher due to the fact that all genes are coded as present/absent (1/0).

*‡ f(correct)* refers to the frequency of correct trees per 100 simulations.

#### ***Simulated data with power-law and non-random distribution of missing data***

For two different sets of genes, differing in relative evolutionary rates among genes (Figure 4), we further simulated 100 (50 taxa × 50 genes) supermatrices each, composed of genes with 400 aa, concatenated for each taxon to 20,000 aa length. We used again the topology derived from empirical data with realistic distribution of branch lengths (Figure 5B). We changed seven branch lengths to introduce potential long branch attraction (Figure 5B). In order to generate supermartices with missing data, we followed a proposal of Li and colleagues [49]. These authors showed that the distribution of missing data in many empirical supermatrices is best described by applying a power law function of the probability of having data. Following their observation, we assigned to each taxon and gene a probability of having data randomly drawn from *f *(*x*) = (1/10 *x*^-1/2^) - 0.1, for *x* randomly selected with equal probability from, 0 ≤ *x* ≤ *∞*. Additionally, we constrained data assignment to having at least one gene for each taxon. Following this approach, we concatenated supermatrices with a distribution of missing data approximately similar to observed empirically supermatrices (Misof, unpubl.) (average data coverage 0.13, Table 1). Finally, we raised the probability of data coverage for four predefined taxa, mimicking the often seen high coverage of a few taxa for which genomes are available.

### Selecting subsets from simulated data and tree reconstructions

#### ***Selecting subsets with the hill climbing algorithm***

SOS’s were selected using the *mare* software (*mare*: **ma**trix **re**duction) which implements the herein described novel approach. For each supermatrix, trees were reconstructed 1) using the original supermatrix (data coverage 0.3), 2) an SOS of *B *and 3) an SOS of *B*^∗^. Trees were reconstructed with RAxML 7.0.0 [50,51]. The BLOSUM62 amino acid substitution matrix with *Γ* distributed among site rate heterogeneity was used to account for different substitution rates among genes.

To compare reconstructed trees with the correct trees used in data simulations, we used standardized quartet distances between shared taxa [24,52-55]. QDistances (*d*_*QD*_) were standardized in relation to all quartets of shared taxa. We recorded *d*_*QD*_’s of trees inferred from the unreduced matrix and of the two SOS’s derived from *B* and *B*^∗^.

#### ***Selecting subsets with predefined thresholds of data coverage***

From supermatrices with power-law and non-random distribution of missing data we selected subsets in two different ways: (1) we selected all genes with data coverage above or equal to 0.4 and (2) we selected all taxa with data coverage above or equal to 0.04 and all genes with data coverage above or equal to 0.4 (adapted to the new number of taxa). We recorded *d*_*QD*_’s of trees inferred from unreduced matrices and from subsets.

### Selecting subsets from empirical data and tree reconstructions

We studied the performance of using the hill climbing algorithm with matrices *B* and *B*^∗^ using the published empirical metazoan data set of Driskell et al. [2] comprising 1,131 putative orthologous genes for 70 taxa (Metazoa, Fungi + outgroup). Additionally, we selected data subsets of the Driskell supermatrix applying predefined thresholds of gene - and taxa coverage (Table 2). All ML analyses using RAxML v7.2.6 or 7.2.8 were executed with rapid bootstrapping (PROTCAT) and best tree search (PROTGAMMA) in one step (-f a, 500 or 1,000 BS replicates) and the empirical substitution matrix WAG [56]. *A posteriori* bootstop tests were performed to test for a sufficient number of bootstrap replicates [57]. All analyses were conducted using RAxML HYBRID and PTHREADS versions on HPC Linux clusters, 8 nodes with 8 or 12 cores each, at the Regionales Rechenzentrum Köln (RRZK) using **C**ologne **H**igh **E**fficient **O**perating **P**latform for **S**cience (CHEOPS). Further, we compared the effects of data reduction on tree robustness with the resolution score as introduced by Holland and colleagues [58]. This resolution score, *RS*, calculated as the sum of bootstrap support values ≥50 divided by the number of taxa *N *- 3, represents a measure of average bootstrap support and, thus, robustness of trees.

**Table 2:** **Comparison of matrix reductions with empirical data using *mare *** **and simple predefined thresholds**

**Data**	**Reduction**	**Number of taxa**	**Resolution score**
Original	Unreduced	70	91.0896
Without 6genome taxa	Unreduced	64	82.1475
	*mare *–t 1.67	48	87.3778
*mare *with *B*^∗^	Default	12	99.5556
	–t 3	13	100
	–t 4	20	94
	–t 6	22	95.8421
	–t 7	26	96.1739
	All taxa constraint	69	87.3485
*mare *with *B*	Default	13	99.5
	–t 3	15	99.8333
	–t 4	21	95.0556
	–t 6	66	88.5238
	–t 7	67	88.4375
	All taxa constraint	69	85.803
Simple thresholdsof coverage	Genes 0.4, taxa 0.4	22	92.5263
	Taxa 0.66, genes 0.66	26	82.2174
	Genes 0.4	59	90.1071
	Genes 0.66	57	80.8704

## Results

### Performance with simulated data

**Figure 7:**
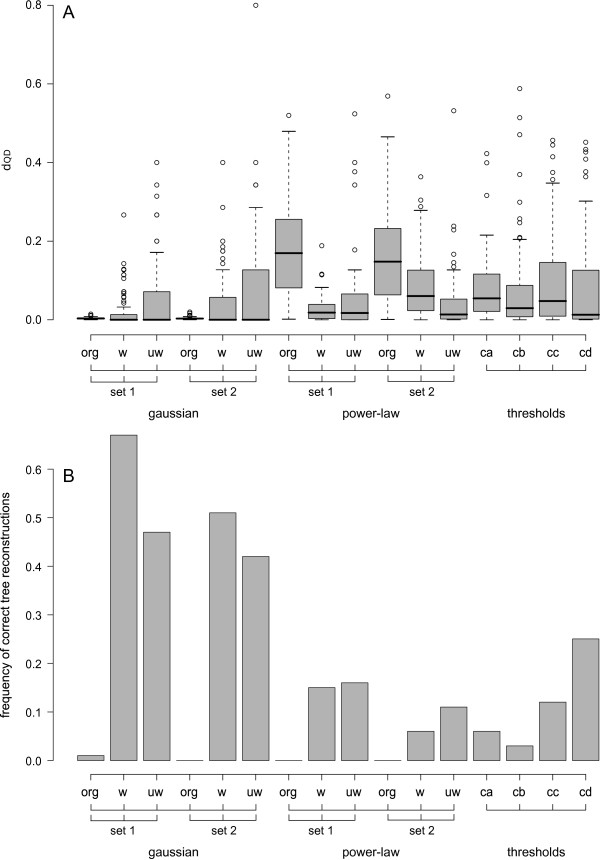
**Summary of results of tree reconstructions of the four different set ups.** In this figure, columns (org) refer to tree reconstructions based on unreduced matrices, columns (w) refer to tree reconstructions based on reduced weighted matrices *B*^∗^, columns (uw) refer to tree reconstructions based on reduced unweighted matrices *B*, columns (ca, cb) refer to tree reconstructions based on the application of thresholds to genes only and columns (cc, cd) refer to tree reconstructions based on the application of thresholds to genes and taxa concerning data coverage. Set1 refers to datasets with relatively low among gene rate heterogeneity and set 2 refers to datasets with relatively high among gene rate heterogeneity. Columns labeled (gaussian) illustrate results derived from datasets with gaussian distribution of missing data, columns labeled (power-law) illustrate results derived from datasets with power-law and non-random distribution of missing data and columns labeled (thresholds) illustrate results derived from datasets reduced by applied predefined thresholds of data coverage. In **(A)** the distribution of *d*_*QD *_values for each simulation and set up is presented as box plots with median (black bar), quartiles (box), whiskers (doted range) and outliers (circles). The smaller *d*_*QD*_, the more similar is the reconstructed tree to the tree used in data simulations. In **(B)** columns represent the frequency of correct tree reconstruction for each set up.

Tree reconstructions based on unreduced supermatrices with a Gaussian distribution of missing data did not yield correct trees except for one case in set 1 (columns (org) for set1 and set2, Gaussian distribution of missing data in Figure 7A,B, Table 1). The variability of *d*_*QD *_values was low (columns (org) for set1 and set2, Gaussian distribution of missing data in Figure 7A, Table 1). Tree reconstructions based on all SOSs (unweighted and weighted reductions of set1 and set2) of these supermatrices performed much better (columns (w), (uw) for set1 and set2, Gaussian distribution of missing data in Figure 7A,B, Table 1). Compared with trees derived from unreduced supermatrices, SOSs supported more often correct trees, but had a higher frequencies of wrong quartets (columns (w), (uw) for set1 and set2, Gaussian distribution of missing data in Figure 7A,B, Table 1). However, there was no clear difference of mean *d*_*QD *_values between trees based on SOSs derived from *B* (uw) or *B*^∗^ (w) (columns (w), (uw) for set1 and set2, Gaussian distribution of missing data in Figure 7A, Table 1). Trees based on SOSs of *B*^∗^ (w) had a much lower amplitude of *d*_*QD *_values (columns (w), (uw) for set1 and set2, Gaussian distribution of missing data in Figure 7A, Table 1). SOSs derived from *B*^∗^ contained on average more taxa (Table 1).

Tree reconstructions based on the unreduced matrix with power-law non-random distribution of missing data did not recover correct trees for set 1 and set 2. In both cases variability of *d*_*QD *_values was high (columns (w), (uw) for set1 and set2, power-law non-random distribution of missing data in Figure 7A,B, Table 1). Tree reconstructions based on all SOSs (unweighted and weighted reductions of set 1 and set2) clearly outperformed reconstructions based on the unreduced matrices (columns (org), (w), (uw) for set1 and set2, power-law non-random distribution of missing data in Figure 7A,B, Table 1). The absolute number of correct trees was again higher for all SOSs (unweighted and weighted reductions of set 1 and set2) compared with the number of correct trees inferred from the unreduced matrices. In cases of low relative rate differences among genes, set 1, SOSs derived from *B* (uw) performed worse compared to SOSs derived from *B*^∗^ (w), in cases of high relative rate differences among genes, set 2, the opposite was observed (columns (org), (w), (uw) for set1 and set2, power-law non-random distribution of missing data in Figure 7B, Table 1).

Data subsets derived from matrices with power-law non-random distribution of missing data using predefined thresholds of gene coverage supported trees with lower mean *d*_*QD *_values (columns (ca), (cb) in Figure 7A) in comparison with mean *d*_*QD*_ values of trees inferred from SOSs selected with our approach (column (w), (uw) for set 1 and set 2 of the power-law data in Figure 7A, Table 1). The mean *d*_*QD *_values were higher and the amplitude of *d*_*QD *_was large (columns (ca), (cb) in Figure 7A). Data subsets from matrices with power-law non-random distribution of missing data using combined thresholds of data coverage for genes and taxa did support trees with mean *d*_*QD *_values (columns (cc), (cd) in Figure 7A) comparable with mean *d*_*QD *_values of trees inferred from SOSs of set 1 and set 2 selected with our approach (column (w), (uw) for set 1 and set 2 of the power-law data in Figure 7A, Table 1). The amplitude of *d*_*QD *_values however was large (columns (cc), (cd) in Figure 7A). Applying only thresholds for gene data coverage yielded a lower absolute number of correct trees (columns (ca), (cb) in Figure 7B) compared with our approach, but the absolute number of correct trees was comparable or even higher if combined thresholds of taxa and genes were used (columns (cc), (cd) in Figure 7, Table 1).

In summary, reduction of supermatrices often increased the chance to find a correct tree, but not consistently. SOSs derived from *B*^∗^ did not always support correct trees more often compared with SOSs derived from *B*, but had a much smaller amplitude of *d*_*Q *_values. Data subsets derived from predefined thresholds supported fewer correct trees if only applied to genes but supported comparable numbers of correct trees if used with combined thresholds of data coverage for taxa and genes.

### Performance with empirical data

We applied our approach to the published metazoan data set of Driskell et al. [2] comprising 1,131 genes for 70 taxa (Metazoa, Fungi + outgroup). The data coverage was low (0.0836), the matrix information content was low (*P *= 0.0657). Most genes are represented only by few taxa (e.g. *Homo sapiens*, *Mus musculus*, *Rattus norvegicus*, *Bos taurus*, *Sus scofra*). We excluded six taxa of which the complete genome was available from the original matrix showing the highest coverage (*Homo sapiens*, *Mus musculus*, *Rattus norwegicus*, *Sus scofra*, *Bos taurus*, and *Gallus gallus*) and selected an SOS from these data. With this procedure we removed the most extreme heterogeneity of data coverage among taxa prior to the selection of an SOS.

Selecting an SOS resulted in a data subset of 48 taxa and 45 genes with a data coverage of 0.316 and *P *= 0.223. Thus, a SOS was found with a 10.24% loss of taxa and a 9.08-fold increase in data coverage and a 16.043-fold gain in *P*. However, all outgroup taxa including slime molds, fungi and nematodes had been excluded. We compared tree reconstructions based on 1) the original unreduced supermatrix with 64 taxa (1000 bs replicates, 469,480 aa) and 2) the SOS of 48 taxa and 45 genes (1,000 bs replicates, 11,198 aa). An *a posteriori* bootstop test (default MR-based bootstopping criterion, WRF average of 100 random splits) revealed that 1,000 BS were by far sufficient for both analyzed data sets.

**Figure 8:**
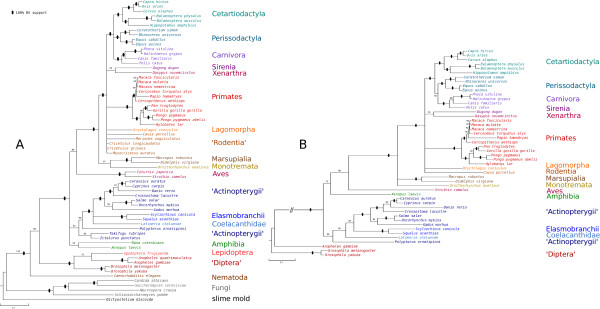
**Driskell trees.** Tree reconstructions based on the **(A)** 64-taxa set and on **(B)** the reduced data. Nodes with less than 50% are collapsed. Black dots: 100% BS support.

Tree reconstructions with the 64-taxa set resulted in trees with polyphyletic Tetrapoda, Actinopterygii, monophyletic Marsupialia + Monotrema, and largely unresolved basal splits within Theria (Figure 8A).

The tree based on the SOS was more congruent to general taxonomic views. The topology showed moderately supported monophyletic Tetrapoda, and resolution within Ungulates and Carnivora (Figure 8). However, for example Actinopterygii remained paraphyletic and relationships of Marsupialia and Monotrema were not resolved. The resolution score *RS* increased from 82.148% (unreduced supermatrix including 64 taxa and 1,1131 genes) to 87.38% (SOS). We also compared reductions of the original Driskell supermatrix using different parameter settings in our approach and simple thresholds of data masking (Table 2). Applying predefined thresholds of gene and taxa coverage never resulted in matrices with comparable resolution scores and comparable number of taxa. Our approach outperformed the application of simple thresholds.

## Discussion

We show that supermatrices of simulated amino acid sequence data with low data coverage and relative rate differences among genes can support biased tree inference or low robustness of trees. It can be suspected that these effects will even be stronger for empirical data. These conclusions corroborate results of Hartmann [24], in many aspects Philippe [22] and Wiens and colleagues [28]. Effective techniques to reduce these potential biases in tree inference are therefore clearly needed.

Masking supermatrices and deleting rogue taxa after tree reconstructions could be suitable measures as has been applied by Dunn and colleagues [4]. In their analysis these authors selected taxa and genes according to predefined cutoff values of data coverage. The application of cutoff values considers only the extent of missing data which might favor the selection of the most conserved genes readily identified among all taxa in the data. Additionally, Dunn et al. [4] deleted rogue taxa after tree reconstruction based on an idea introduced by Thorley and colleagues [59,60]. The major drawback of their approach is that robustly misplaced taxa will not be identified. In this respect, a formal approach to masking of supermatrices as proposed here could be an alternative worth to consider.

We propose to select a subset of taxa and genes with a maximal information content. In doing so, it is necessary to first assess potential signal of genes, for which we use extended geometry mapping (eGM) [37-40]. We opted for geometry mapping, because it tends to be more conservative in discriminating between resolved and star-like trees in contrast to likelihood mapping [61]. Additionally, eGM is easily applied to nucleotide and amino acid sequence data without the need of tree reconstructions. It is, thus, a technically convenient but, admittedly, coarse way of estimating potential signal.

Secondly, it is necessary to select optimal subsets of supermatrices based on the information content of taxa and genes. The information content of taxa and genes is calculated as the ratio of potential signal and data coverage. By introducing this optimality criterion we can select taxa and genes which contribute most signal in tree reconstructions. We select a data subset in a stepwise function penalizing size reduction of the supermatrix and favoring higher matrix information content, monitoring but ignoring optimization of connectivity in the matrix. Our approach is time efficient but will not be effective in discovering a globally optimal subset in terms of taxa/gene overlap (‘connectivity’) and information content. This is in contrast to the approach of Yan [44] in which the *quasi-biclique* with the highest level of connectivity (‘largest grove’) is searched for.

Improved heuristics considering information content *and * connectivity in our approach are certainly conceivable. However, the distribution of missing data following a power-law distribution in empirical data suggests that simple hill climbing procedures will be effective in identifying a good (optimal) subset of taxa and genes in terms of matrix information content. The flexibility of our approach offers even the chance to use different parameter settings of the optimality function to identify alternative SOSs.

We observed high amplitudes of *d*_*QD *_values of trees based on SOSs in our simulations. These amplitudes were even higher in SOS’s based on simple data coverage representations. We interpret this occasional high error rate as a possible phenomenon of insufficient taxon sampling in SOSs which might pronounce long branch attraction (LBA), or, alternatively, that connectivity in SOSs was not sufficient to potentially support just one tree [62]. This interpretation highlights a problem of all methods of data reduction. Every reduction process, at least partially, counteracts efforts to reduce biases in tree reconstructions due to insufficient taxon or gene sampling. The analyses of Wiens and colleagues [20,21,28] showed that LBA effects can disappear, if data exhibiting LBA are recoded as missing. This implies that an identification of LBA taxa before concatenation and reduction of data would be important. However, we do not have a grip yet on a reliable identification of biases in tree reconstructions which could guide a preselection of taxa. An immediate, however unsatisfying, solution is probably the reconstruction of trees with and without suspect taxa.

Our simulations showed that in the presence of heterogeneous signal among genes the new heuristics increased the chance of finding a correct tree. It is, thus, an alternative to the computationally much more demanding *quasi-biclique* approach [44,45]. SOSs derived from *B* or *B*^∗^ matrices did not differ extensively in their success rate of correct tree reconstructions with simulated data, with small advantages for the *B* in cases of power-law non-random distribution of missing data. However, the analyses of the empirical data imply that tree reconstructions based on SOSs derived from *B*^∗^ will result in improved tree robustness.

## Conclusions

Our analyses of simulated and empirical data demonstrate that sparse supermatrices can be reduced on a formal basis outperforming the usually used simple selections of taxa and genes with high data coverage. The approach prresented here is will be of general inportance in phylogenomic studies based on large concatenated superalignments with incomplete data coverage. It clearly offers an alternative to threshold based data selection.

## Competing interests

The authors declare that they have no competing interests.

## Authors’ contributions

B.Mi., B.Me. conceived the study, designed the setup and performed all analyses. B.Mi. wrote the paper with comments and revisions from K.Me., K.Mi., P.K., B.v.R. and B.Me. All authors read and approved the final manuscript.
